# Supplementary material for: Adlay Consumption Combined with Suspension Training Improves Blood Lipids and Pulse Wave Velocity in Middle-Aged Women
Source: Healthcare (Basel). 2023 May 15;11(10):1426. doi: 10.3390/healthcare11101426 (PMC10218412; doi:10.3390/healthcare11101426)
Supplement: Supplementary file 1 [file healthcare-11-01426-s001.zip › healthcare-2232677-supplementary.pdf]

Table S1. Changes in body composition with adlay and suspension training during 12weeks by middle-aged women

Variables	Group	Pre	Post	Post-hoc
BMI (kg/m^2)	ASEG	22.4±2.3	22.1±2.2	ASEG<CON<br>SEG<CON
	SEG	22.1±3.0	21.8±2.6	
	CON	22.1±2.9	22.5±3.0	
LBM (kg)	ASEG	40.6±2.7	41.6±2.4	-
	SEG	39.5±3.0	40.1±2.4	
	CON	40.3±3.0	40.5±3.2$^{\$}$	

Values are presented as mean±standard deviation. ASEG: Adlay+Suspension Exercise Group, SEG: Suspension Exercise Group, CON: Control.
*p<.05, **p<.01; $^{\$}$p<.05 vs pre

Table S2. Changes in Physical Fitness with adlay and suspension training during 12weeks by middle-aged women

Variables	Group	Pre	Post	Post-hoc
Grip strength (kg)	ASEG	27.3±2.9	29.4±2.8$^{\$\$}$	SEG<ASEG<br>CON<SEG
	SEG	27.4±3.0	28.0±3.0$^{\$}$	
	CON	27.7±2.8	27.7±2.9	
Sit-ups (rep/60sec)	ASEG	24.1±6.5	29.1±7.8$^{\$\$}$	CON<ASEG<br>CON<SEG
	SEG	24.5±5.7	27.4±5.8$^{\$\$}$	
	CON	24.1±10.3	23.8±9.8	
Sit&Reach (cm)	ASEG	8.5±3.2	9.1±3.0	-
	SEG	8.5±3.6	8.8±3.8	
	CON	8.6±3.1	8.9±3.5	
20m multi stage (laps)	ASEG	19.1±4.1	21.8±4.1$^{\$\$}$	CON<ASEG<br>CON<SEG
	SEG	19.8±6.5	21.4±6.4$^{\$}$	
	CON	19.4±4.8	19.1±4.9	

Values are presented as mean±standard deviation. ASEG: Adlay+Suspension Exercise Group, SEG: Suspension Exercise Group, CON: Control.
*p<.05, **p<.01; $^{\$}$p<.05, $^{\$\$}$p<.01 vs pre

Table S3. Changes in Blood lipids with adlay and suspension training during 12weeks by middle-aged women

Variables	Group	Pre	Post	Post-hoc
	ASEG	177.1±11.8	175.3±12.4	
TC (mg/dL)	SEG	181.0±18.1	179.9±17.3	-
	CON	179.1±12.8	180.5±12.3	
	ASEG	80.6±8.8	77.8±9.7$^{\$\$}$	
TG (mg/dL)	SEG	81.5±9.2	80.4±8.2	-
	CON	80.3±11.3	80.4±14.6	
	ASEG	50.3±6.0	53.6±5.8$^{\$}$	
HDL-C (mg/dL)	SEG	51.8±4.8	53.8±4.4	-
	CON	51.9±6.0	51.6±5.0	
	ASEG	115.6±6.8	110.6±7.8$^{\$\$}$	
LDL-C (mg/dL)	SEG	115.3±8.3	112.5±7.8	ASEG<CON
	CON	115.0±9.5	114.4±8.6	

Values are presented as mean±standard deviation. TC: Total Cholesterol, TG: Triglyceride, HDL-C: High Density Lipoprotein Cholesterol, LDL-C: Low Density Lipoprotein Cholesterol, ASEG: Adlay+Suspension Exercise Group, SEG: Suspension Exercise Group, CON: Control.
$^{*}p<.05$, $^{**}p<.01$; $^{\$}p<.05$, $^{\$\$}p<.01$ vs pre

Table S4. Changes in Pulse wave velocity with adlay and suspension training during 12weeks by middle-aged women

Variables	Group	Pre	Post	Post-hoc
	ASEG	8.3±0.7	7.3±0.7$^{\$\$}$	
PWV (sec)	SEG	8.3±0.6	8.2±0.6	ASEG<SEG<br>ASEG<CON
	CON	8.2±0.7	8.3±0.7	

Values are presented as mean±standard deviation. PWV: Pulse Wave Velocity, ASEG: Adlay+Suspension Exercise Group, SEG: Suspension Exercise Group, CON: Control.
$^{*}p<.05$, $^{**}p<.01$; $^{\$\$}p<.01$ vs pre
